# Post-Diagnostic Dietary and Lifestyle Factors and Prostate Cancer Recurrence, Progression, and Mortality

**DOI:** 10.1007/s11912-021-01017-x

**Published:** 2021-03-10

**Authors:** Crystal S. Langlais, Rebecca E. Graff, Erin L. Van Blarigan, Nynikka R. Palmer, Samuel L. Washington, June M. Chan, Stacey A. Kenfield

**Affiliations:** 1 Department of Epidemiology & Biostatistics, University of California, San Francisco, 550 16th Street, San Francisco, CA USA; 2 Helen Diller Family Comprehensive Cancer Center, University of California, San Francisco, San Francisco, CA USA; 3 Department of Urology, University of California, San Francisco, San Francisco, CA USA; 4 Department of Medicine, Division of General Internal Medicine at Zuckerberg San Francisco General Hospital, University of California, San Francisco, San Francisco, CA USA

**Keywords:** Nutrition, Physical activity, Exercise, Survivorship, Modifiable risk factors, Prostate cancer, Fish, Meat, Poultry, Eggs, Dairy, Dietary fats, Cruciferous vegetables, Tomatoes, Alcohol, Supplements, Obesity, Body mass index, Smoking

## Abstract

**Purpose of Review:**

This study aimed to summarize evidence published between 1999 and June 2020 examining diet and lifestyle after prostate cancer (PC) diagnosis in relation to risk of biochemical recurrence, PC progression, and PC-specific mortality.

**Recent Findings:**

Secondary prevention is an important research area in cancer survivorship. A growing number of studies have reported associations between post-diagnostic modifiable behaviors and risk of PC outcomes.

**Summary:**

Evidence on modifiable lifestyle factors and PC remains limited. Where multiple studies exist, findings are often mixed. However, studies consistently suggest that smoking and consumption of whole milk/high-fat dairy are associated with higher risk of PC recurrence and mortality. In addition, physical activity and ½ to 1 glass of red wine/day have been associated with lower risk of recurrence and PC-specific mortality. Greater inclusion of racially/ethnically diverse groups in future research is necessary to understand these relationships in populations most impacted by adverse PC outcomes.

## Introduction

Prostate cancer (PC) is the second most common malignancy diagnosed among men worldwide, with an estimated 1.3 million diagnoses worldwide in 2018 [1]. Despite its relatively high survival rate, it remains the fifth most common cause of cancer-related death among men worldwide, with 358,989 deaths reported in 2018. Moreover, it is the leading cancer-related cause of death in men in 46 countries [1]. The varying disease courses PC can take underscore its heterogeneity in presentation and prognosis and highlight the importance of secondary prevention. Over the past two decades, there has been a growing interest in identifying modifiable factors, such as diet and lifestyle factors, associated with overall health, disease progression, and mortality among men with PC.

## Methods

In this review, we summarize findings from studies evaluating associations of *post-diagnosti*c dietary and lifestyle (e.g., physical activity, body size, smoking) behaviors with PC recurrence, progression, and mortality; highlight important new research; and discuss where additional research is needed. We focused on observational studies to complement a recent review of randomized trials on this topic [2•]. Although focused on literature from the last 5 years, additional studies from the past two decades provide further context. We used the search terms “prostate cancer”, “progression”, and “mortality” in combination with each dietary or lifestyle factor (see subsection headers below) to search PubMed for articles published through June 22, 2020. Papers that examined all-cause mortality (ACM) were included if a PC-specific outcome (recurrence/progression, PC-specific mortality (PCSM)) was also evaluated. A single author (CSL) reviewed titles and abstracts of 1894 returns and identified 168 unique articles for further review. Eighty-three were deemed relevant, 33 of which were published between 2015 and 2020. Most common reasons for exclusion included exposure assessment prior to diagnosis and lacking assessment of any of the outcomes of interest.

Given known racial/ethnic disparities in PC, including a greater mortality burden among African-American/Black (AA/B) men, we assessed the race/ethnicity distributions of the populations studied. Table 1 summarizes characteristics of the studies reviewed, stratified by exposure. Table 2 summarizes the findings of all included observational studies. Where relevant, we supplement our discussion with findings from randomized trials [2•].

**Table 1:** Characteristics of studies by diet and lifestyle factor

Author, year	Country	Population	Disease status	*N*	Race/ethnicity	Age range and mean/median	Specific exposure(s) examined	Outcome(s)^a^
**Fish**								
Wang, 2020 [3]	USA	CPS-II Nutrition Cohort	Non-metastatic	4882	White = 98%^b^ AA/B = 1%^b^ Other = 1%^b^	NR^c^	Fish	PCSM, ACM
Wilson, 2016 [4]	USA	Washington University Genetics Study	≤ T3 and RP as primary TX	940	White: 96% Other NR	Mean: 61 Range: NR	Fish, fried fish, not-fried fish	Recurrence
Yang, 2015 [5]	USA	PHS I and II	Non-metastatic	926	White: 96% Other NR	Mean: 69 Range: NR	Fish	PCSM, ACM
Richman, 2010 [6]	USA	CaPSURE	Localized or regional	1294	White: 96%^b^ AA/B: 3%^b^ Other: 1%^b^	NR^c^	Fish	Progression
Chan, 2006 [7]	USA	HPFS	Localized or regional	1202	White: > 95%^d^ AA/B: 1% Other: NR	Mean: 68 Range: NR	Fish	Progression
**Meat, Poultry, Eggs**								
Wang, 2020 [3]	USA	CPS-II Nutrition Cohort	Non-metastatic	4882	White = 98%^b^ AA/B = 1%^b^ Other = 1%^b^	NR^c^	Total red and processed meat, unprocessed red meat, processed meat, poultry, unprocessed poultry, eggs	PCSM, ACM
Wilson, 2016 [4]	USA	Washington University Genetics Study	≤ T3 and RP TX	940	White: 96% Other NR	Mean: 61 Range: NR	Total red meat, unprocessed red meat, processed meat, rare/medium rare red meat, well/very well-done red meat, poultry, fried poultry, not-fried poultry, eggs	Recurrence
Yang 2015 [5]	USA	PHS I and II	Non-metastatic	926	White: 96% Other NR	Mean: 69 Range: NR	Processed meat, red meat, eggs	PCSM, ACM
Richman, 2011 [8]	USA	HPFS	Localized or regional	3127	White: 92%^b^ Other NR	NR^c^	Total red meat, unprocessed red meat, processed red meat, total poultry, eggs, hamburger, beef/lamb/pork sandwich or mixed dish, beef/lamb/pork main dish, sausage/salami/ bologna, bacon, hot dogs, chicken/ turkey with and without skin, chicken/turkey sandwiches, chicken/turkey hot dogs	Lethal PC
Richman, 2010 [6]	USA	CaPSURE	Localized or regional	1294	White: 96%^b^ AA/B: 3%^b^ Other: 1%^b^	NR^c^	Processed red meat, unprocessed red meat, poultry, poultry with skin, skinless poultry, eggs	Progression
Chan, 2006 [7]	USA	HPFS	Localized or regional	1202	White: > 95%^d^ AA/B: 1% Other: NR	Mean: 68 Range: NR	Red meat	Progression
**Dairy**								
Tat, 2018 [9]	USA	CaPSURE	Non-metastatic	1334	White: 96%^e^ AA/B: 2%^e^ Other: 2%^e^	NR^c^	Whole milk, skim/low-fat milk, total dairy, high-fat dairy, low-fat dairy, ice cream, yogurt, butter, cream, sherbet, cottage cheese, cream cheese, other cheese	Progression
Downer, 2017 [10]	Sweden	Population-based	Any	525	NR	Mean: 71 Range: NR	Total dairy, high-fat dairy, low-fat dairy, total milk, sour milk, high-fat milk, low-fat milk, butter, cheese	PCSM, ACM
Yang, 2015 [5]	USA	PHS I and II	Non-metastatic	926	White: 96% Other NR	Mean: 69 Range: NR	High-fat dairy, butter	PCSM, ACM
Pettersson, 2012 [11]	USA	HPFS	Localized/locally advanced	3918	White: 96%^b^ Other: NR	NR^c^	Total milk, skim-2% milk, whole milk, total dairy, low-fat dairy, full-fat dairy	Progression, lethal PC
Chan, 2006 [7]	USA	HPFS	Localized or regional	1202	White: > 95%^d^ AA/B: 1% Other: NR	Mean: 68 Range: NR	Milk	Progression
**Dietary Fats**								
Van Blarigan, 2015 [12]	USA	PHS I and II	Non-metastatic	926	White: 96%^b^ Other: NR	NR^c^	Saturated fat, monounsaturated fat, polyunsaturated n-6 and n-3 fatty acids, *trans* fat, animal fat, vegetable fat	PCSM, ACM
Richman, 2013 [13]	USA	HPFS	Non-metastatic	4577	White: 93%^b^ Other: NR	NR^c^	Saturated fat, monounsaturated fat, polyunsaturated fat, trans fat, animal fat, vegetable fat	Lethal PC, ACM
Epstein, 2012 [14]	Sweden	Population-based	Any	525	NR	NR^c^	Total fat, saturated fat, monounsaturated fat, polyunsaturated omega-6 and omega-3 fatty acids^f^	PCSM
Strom, 2008 [15]	USA	MD Anderson Cancer Center	RP TX	390	White: 100%	Mean: 61^e^ Range: NR	Saturated fat	Recurrence
Meyer, 1999 [16]	Canada	Population-based	Any	384	NR	Mean: 67 Range: NR	Total fat, saturated fat, monounsaturated fat, polyunsaturated fat	PCSM
**Vegetables: Tomato (Lycopene), Cruciferous**								
Yang, 2015 [5]	USA	PHS I and II	Non-metastatic	926	White: 96% Other: NR	Mean: 69 Range: NR	Cruciferous vegetables, tomato	PCSM, ACM
Richman, 2012 [17]	USA	CaPSURE	Non-metastatic (< T3b)	1560	White: 95%^e^ AA/B: 3%^e^ Other: 2%^e^	< 60: 26%^e^ 60–69: 45%^e^ ≥ 70: 29%^e^	Cruciferous vegetables, broccoli, cabbage, cauliflower, Brussels sprout, kale, tomato sauce, tomato	Progression
Chan, 2006 [7]	USA	HPFS	Localized or regional	1202	White: > 95%^d^ AA/B: 1% Other: NR	Mean: 68 Range: NR	Tomato sauce, tomato	Progression
**Alcohol**								
Downer, 2019 [18]	USA	HPFS	Non-metastatic	5182	White: 92%^e^ Other: NR	Mean: 70^e^ Range: NR	Total alcohol, total wine, beer, liquor, red wine, white wine	Lethal PC, ACM
Farris, 2018 [19]	Canada	Population-based	≥ T2	829	NR	Mean: 67 Range: NR	Total alcohol, beer, liquor, wine	Recurrence, PCSM, ACM
**Supplements**								
Nair-Shaliker, 2020 [20]	Australia	New South Wales PC Care Outcomes Study		1119	Australian: 78%^g^ Other: 22%^g^	65–85: 34% 60–64: 29% 55–59: 24% 49–54: 14%	Serum 25 OHD, serum 1,25(OH)_2_D	PCSM, ACM
Downer, 2017 [10]	Sweden	Population-based	Newly diagnosed	525	NR	Mean: 71 Range: NR	Calcium, phosphorous, vitamin D	PCSM, ACM
Kenfield, 2015 [21]	USA	HPFS	Non-metastatic	4459^h^	White: > 95%^d^ Other: NR	Mean: 69^e^ Range: NR	Selenium supplement use	Recurrence, PCSM, ACM
Holt, 2013 [22]	USA	Seattle-Puget Sound Tumor Registry	Newly diagnosed	1476	White: 90%^e^ AA/B: 10%^e^	Mean: 60^e^ Range: NR	Serum 25(OH) D	Recurrence/progression, PCSM
**Obesity**^i^								
Jackson, 2020 [23]	Jamaica	PROSCARE Study	Any	242	African-Caribbean: 100%	Mean: 68 Median: 69 Range: NR	BMI, waist circumference, waist-to-hip ratio	PCSM, ACM
Troeschel, 2020 [24]	USA	CPS-II Nutrition Cohort	Non-metastatic	8330	White: 97% Other: 3%	≥ 80: 6% 75 to < 80: 20% 70 to < 75 32% 65 to < 70: 28% < 65: 13%	BMI	PCSM, ACM
Leal-Garcia, 2020 [25]	Mexico	Multi-institution	RP TX	180	Mexican: 100%	Median: NR^c^ Range: 45–79	BMI	BCR
Vidal, 2020 [26]	USA	SEARCH	RP TX	5929	White: 67% AA/B: 33%	Median: 63 (White) 60 (AA/B) Range: NR	BMI	BCR, PCSM
Langlais, 2019 [27]	USA	CaPSURE	Non-metastatic and RP TX	5200	White: 88%^e^ AA/B: 9%^e^ Other: 3%^e^	Mean: 61^e^ Range: NR	BMI	Recurrence, ACM
Freedland, 2019 [28]	USA	SEARCH	RP TX	4123	White: 59%^e^ AA/B: 38%^e^ Other: 3%^e^	NR^c^	BMI	BCR
Farris, 2018 [29]^j^	Canada	Population-based cohort Population-based cohort	≥ T2 ≥ T2 and survived 2 years post-DX	987 829	NR	NR	BMI, waist circumference, waist-to-hip ratio	PCSM, ACM
Vidal, 2017 [30]	USA	SEARCH	RP TX	4268	White: 59%^e^ AA/B: 37%^e^ Other: 3%^e^	Mean: 62^e^ Range: NR	BMI	BCR, PCSM
Dickerman, 2017 [31]	USA	HPFS	Localized (T1-T2)	5158	White: 97%^e^ Other: NR	Mean: 70^e^ Range: NR	BMI	Recurrence, lethal PC
Schiffmann, 2017 [32]	Germany	Martini-Klinik Prostate Cancer Center	RP TX	16,014	NR	Median: 65 Range: NR	BMI	Recurrence
Khan, 2017 [33]	USA	HCaP-NC	Any	647	White: 56%^e^ AA/B: 44%^e^	Mean: 62^e^ 63^e^ (White) 60^e^ (AA/B) Range: NR	BMI	Progression
Wang, 2015 [34]	USA	Fox Chase Cancer Center, Philadelphia, PA	Localized (T1b-T4N0M0) and IMRT TX	1442	NR	≥ 80: 5% 70–79: 37% 60–69: 41% 36–59: 17%	BMI	Recurrence, distant Mets, PCSM, ACM
**Physical Activity**								
Bonn, 2019 [35]	Sweden	PROCAP study	Localized	4595	NR	Mean: 63 Range: NR	Time spent sitting during leisure time	PCSM, ACM
Dai, 2019 [36]	USA	Population-based	Localized	1354	White: 92%^e^ AA/B: 8%^e^ Other: NR	NR^c^	Vigorous PA	Lethal PC
Guy, 2018 [37]	Canada	Sunnybrook Cohort and Royal Marsden Hospital AS Cohort^m^	Low-intermediate risk and AS	237	White: 86%^k^ AA/B: 7%^k^ Other: 6%^k^ Unknown: 1%^k^	Mean: 65^e^ Range: NR	Total PA, recreational PA, vigorous PA	Reclassification
Wang, 2017 [38]	USA	CPS-II Nutrition Cohort	Non-metastatic	5319	NR^l^	NR^l^	Total recreational PA, other recreational PA, walking	PCSM, ACM
Friedenreich, 2016 [39]	Canada	Alberta Cancer Registry	T 2–4	830	White: 95% Other: 5%	Median: 68 Range: NR	Total PA, recreational PA, non-sedentary occupational PA, household PA, vigorous PA, occupational sedentary behavior	PCSM, ACM
Vandersluis, 2016 [40]	Canada	Sunnybrook Cohort and Royal Marsden Hospital AS Cohort^m^	Low-intermediate risk and AS	237	White: 86%^e^ AA/B: 7%^e^ Other: 6%^e^ Unknown: 1%^e^	Mean: 65^e^ Range: NR	Total PA	Progression
Bonn, 2015 [41]	Sweden	PROCAP study	Localized	4623	NR	Mean: 63 Range: NR	Total recreational PA, walking or biking, household work, exercising	PCSM, ACM
Richman, 2011 [42]	USA	CaPSURE	Localized	1455	NR	Mean: 65 Range NR	Vigorous PA, non-vigorous PA, walking duration, walking pace	Progression
Kenfield, 2011 [43]	USA	HPFS	Non-metastatic	2705	NR	Mean: 69^e^ Range: NR	Total PA, vigorous PA, non-vigorous PA	PCSM, ACM
**Smoking**								
Riviere, 2020 [44]	USA	Veteran Affairs Health Systems	Any	73,668	White: 66%^e^ AA/B: 28%^e^ Other: 6%^e^	NR^c^	Smoking status (current, former, never)	PCSM, ACM
Sato, 2017 [45]	Japan	Harasanshin Hospital, Kyushu University Hospital	RP without neoadjuvant or adjuvant therapy	1165	NR	NR^c^	Smoking status (current, never/former)	Recurrence
Steinberger, 2015 [46]	USA	MSKCC	EBRT TX	2095	NR	< 65: 25%^e^ ≥ 65: 72%^e^ Range: NR	Smoking status (current, former, never)	Recurrence, Distant Mets, PCSM
Rieken, 2015 [47]	Austria, USA	Multi-institution	Localized, RP TX	6538	NR	Median: 61 Range: NR	Smoking status (current, former, never), smoking cessation prior to TX	Recurrence
Moreira, 2014 [48]	USA	SEARCH	RP TX	1670	White: 54%^e^ AA/B: 40%^e^ Other: 5%^e^	NR^c^	Smoking status (current, never/former)	Progression, lethal PC, PCSM, ACM
Ngo, 2013 [49]	USA	Stanford RP Database	RP TX	630	NR	Median 63 Range: NR	Heavy smokers (≥ 20 pack-year), light smoker (< 20 pack-year)	Recurrence
Oh, 2012 [50]	South Korea	Seoul National University	RP TX	1165	NR	Mean: 65^e^ Range: NR	Smoking status (current, never/former)	Recurrence
Joshu, 2011 [51]	USA	Johns Hopkins University	RP TX	1416	White: 95%^e^ AA/B: 2%^e^ Other: 3%^e^	Mean: 57^e^ Range: NR	Smoking status (ever, current, former, never)	Recurrence
Gong, 2008 [52]	USA	Population-based	Any	752	White: 94% AA/B: 6%	60–64: 40% 55–59: 34% 40–54: 26%	Smoking status (current, quit < 10 years, quit > 10 years, never)	PCSM
Pantarotto, 2007 [53]	Canada	Ottawa Hospital Regional Cancer Centre	Localized, EBRT TX	434	NR	Median: 69 Range: 46–83	Smoking status (current, former, never)	Recurrence, Lethal PC, PCSM, ACM
Merrick, 2004 [54]	USA	Schiffler Cancer Center	T1b-T3a, Brachytherapy TX	582	NR	Mean: 67 Median: 68 Range: NR	Smoking status (current, former, never)	Recurrence
Pickles, 2004 [55]	Canada	Multi-institution	Localized, EBRT TX	601	NR	NR^c^	Smoking status (current, never)	Recurrence
Oefelein, 2004 [56]	USA	Case Western University	Advanced	222	NR	Mean: 73^e^ Range: NR	Pack-years	Progression, ACM

*AA/B*, African-American/Black; *ACM*, all-cause mortality; *AS*, active surveillance; *BMI*, body mass index; *CaPSURE*, Cancer of the Prostate Strategic Urologic Research Endeavor; *CPS*, Cancer Prevention Study; *DX*, diagnosis; *HCaP-NC*, Health Care Access and Prostate Cancer Treatment in North Carolina; *EBRT*, external beam radiation therapy; *HPFS*, Health Professionals Follow-up Study; *IMRT*, intensity modulated radiation therapy; *MSKCC*, Memorial Sloan Kettering Cancer Center; *NR*, not reported; *PA*, physical activity; *PC*, prostate cancer; *PCSM*, prostate cancer-specific mortality; *PHS*, Physicians’ Health Study; *PROCAP*, Progression in Cancer of the Prostate; *RP*, radical prostatectomy; *PROSCARE*,: Prostate Cancer Risk Evaluation; *TX*, treatment; *SEARCH*, Shared Equal Access Regional Cancer Hospital database; *UK*, United Kingdom; *USA*, United States of America

^a^Lethal disease typically defined as PC metastases or death

^b^Data for full cohort not provided. Estimated from demographics data provided on half (upper and lower quartile), two-fifths (upper and lower quintile), or three-fifths (upper, middle, and lower quintile) of study sample

^c^Overall age of study sample not reported and not enough data available to calculate

^d^Assumed from other HPFS analyses

^e^Data for full cohort not provided, estimated from stratified data reported

^f^Study also considered subtypes of saturated fatty acids (lauric acid, myristic acid, palmitic acid, stearic acid, arachidic acid, shorter chain), monounsaturated fatty acids (palmitoleic acid, oleic acid), omega-3 polyunsaturated fatty acids (alpha-linolenic acid, eicosapentaenoic acid, docosapentaenoic acid, docosahexaenoic acid, combined marine fatty acids), and omega-6 polyunsaturated fatty acids (linoleic acid, arachidonic acid)

^g^Race/ethnicity reported in study as region of birth

^h^Analyses for recurrence outcome only included 3718 men

^i^We reviewed an additional 29 studies published between 2004 and 2014 [57–85]. Results are summarized in Table 2, but study characteristics are not reported here due to space.

^j^Two samples reported in single paper

^k^As reported in Vandersluis [40], which used the same study population

^l^Study looked at both pre- and post-diagnostic physical activity. Demographics only provided for the pre-diagnostic physical activity cohort.

^m^Examined association separately in the Sunnybrook cohort (*n* = 131) and Royal Marsden Hospital cohort (*n* = 106)

**Table 2:** Summary of associations between diet and lifestyle components and prostate cancer progression, prostate cancer-specific mortality, and all-cause mortality

Dietary Factors	Recurrence/progression association			Lethal disease/PCSM association			ACM association		
	Inverse	Null	Positive	Inverse	Null	Positive	Inverse	Null	Positive
Fish, total	[7]^a,b^	[4, 6, 7]^a^			[3, 5]			[3, 5]	
Fried		[4]							
Non-fried		[4]							
Meat, Poultry, Eggs									
Total red meat		[4, 7]			[3, 5, 8]			[5]	[3]
Unprocessed red meat		[4, 6]		[3]	[8]			[3]	
Rare/medium-rare meat		[4]							
Well-done red meat		[4]							
Hamburger					[8]				
Beef/lamb/pork					[8]				
Processed red meat		[4, 6]			[3, 8]	[5]		[3]	[5]
Sausage/salami/bologna					[8]				
Bacon					[8]				
Hot dogs					[8]				
Total poultry		[4, 6]			[3, 8]		[3]		
Chicken/turkey hot dogs					[8]				
Unprocessed poultry^c^					[3]		[3]^d^		
Chicken or turkey, no skin		[6]			[8]				
Chicken or turkey, with skin			[6]		[8]				
Chicken or turkey Sandwiches					[8]				
Fried poultry		[4]							
Non-fried poultry		[4]							
Eggs		[4]	[6]^d^		[3, 5, 8]			[3]	[5]
Dairy									
Total dairy		[9, 11]			[10, 11]			[10]	
High-fat/full-fat dairy		[9, 11]			[5, 10, 11]				[5, 10]^d^
Low-fat dairy		[9, 11]			[10, 11]			[10]	
Total milk		[7, 11]^a^	[7]^a,b^		[10, 11]			[10]	
Sour milk					[10]			[10]	
Whole/high-fat milk			[9, 11]		[10]^e^	[10, 11]^e^		[10]^e^	[10]^e^
Skim/low-fat milk		[9]			[10]			[10]^f^	[10]^f^
Skim-2% milk		[11]			[11]				
Cream		[9]							
Butter		[9]			[5, 10]			[5, 10]	
Ice cream		[9]							
Yogurt		[9]							
Sherbet	[9]^d^								
Cheese					[10]			[10]	
Other cheese		[9]							
Dietary Fats, total					[14, 16]^e^	[14]^e^			
Saturated			[15]		[13, 14]	[12, 16]^b,d^			[12, 13]^b^
Monounsaturated					[12–14, 16]			[12, 13]	
Polyunsaturated^g^					[12–14, 16]		[13]^b^	[12]	
Trans					[12, 13]			[12]	[13]
Animal					[12, 13]			[12, 13]	
Vegetable				[13]^b^	[12]		[12, 13]		
Vegetables: Tomato, Cruciferous									
(Fresh) tomato		[7, 17]^a^	[7]^a^		[5]			[5]	
Tomato sauce	[7]^a^	[7, 17]^a^							
Cruciferous Vegetable, total	[17]				[5]			[5]	
Broccoli		[17]							
Cabbage		[17]							
Cauliflower		[17]							
Brussel sprouts		[17]							
Kale		[17]							
Lifestyle factors	Recurrence/progression association			Lethal disease/PCSM association			ACM association		
	Inverse	Null	Positive	Inverse	Null	Positive	Inverse	Null	Positive
Alcohol, total		[19]			[18, 19]			[18, 19]	
Beer					[19]	[18]^b,d^		[18, 19]	
Liquor					[18, 19]			[18, 19]	
Total wine				[18]^b^	[19]		[19]	[18]	
Red wine				[18]			[18]		
White wine					[18]			[18]	
Supplement use									
Selenium		[21]				[21]		[21]	
Vitamin D		[22]^h^			[10, 20, 22]^h,i^		[20]^j^	[10, 20]^j^	
Calcium					[10]			[10]	
Phosphorous					[10]			[10]	
Obesity									
Body mass index		[25–27, 31–33, 57, 60, 62, 65, 68–70, 77, 79, 83, 85]^h,k,l^	[28, 30, 34, 61, 63, 64, 66, 67, 71–76, 78, 80–82, 84]^d^		[23, 24, 26, 29–31, 57, 60, 65, 68, 79]^h,k,m^	[34, 57–59]^h,m^	[77]^l^	[23, 29, 59, 60, 65, 68, 77, 79]^h,l^	[24, 27, 34]
Waist circumference					[23, 29]^h^			[23, 29]^h^	
Waist-to-hip ratio					[23, 29]^h^			[23, 29]^h^	
Physical Activity, total	[37]	[40]		[43]	[39]		[39, 43]		
Vigorous	[37]	[42]		[36, 43]	[39]		[39, 43]		
Non-vigorous		[42]			[43]		[43]		
Exercising^n^				[41]			[41]		
Recreational		[37]		[38, 39]^p^	[41]		[38, 39, 41]^p^		
Other (non-walking) recreational					[38]		[38]		
Household					[39, 41]		[41]	[39]	
Non-sedentary occupational					[39]		[39]		
Occupational sedentary behavior					[39]			[39]	
Time sitting during leisure time					[35]^h^			[35]^h^	
Walking/walking or biking ^[41]^	[42]^o^	[42]^o^		[41]	[38]		[38, 41]^p^		
Smoking									
Current (vs never)		[53, 54]	[46, 47, 51, 55]		[53]^m^	[44, 46, 52, 53]^m^		[53]	[44]
Current (vs never/former)		[45, 48, 50]^r^	[50]^r^			[48]			[48]
Former (vs never)		[46, 51, 53, 54]	[47]		[44, 46, 52, 53]^m^	[53]^m^		[53]	[44]
Smoking cessation		[47]^s^	[47]^s^						
Heavy^q^ vs light smoker			[49]						
Per pack-year			[56]						[56]

*AA/B*, African-American/Black; *ACM*, all-cause mortality; *BMI*, body mass index; *METs*, metabolic equivalents; *PCSM*, prostate cancer-specific mortality

^a^Study [7] observed no association with progression without adjustment for, and a statistically significant association with adjustment for, pre-diagnostic intake

^b^Studies [7, 12, 13, 18] evaluated exposure as categorical and continuous variables, only reached statistical significance in one of these models

^c^Study [3] only examined unprocessed poultry among subgroup of 3344 men without known history of cardiovascular disease

^d^Borderline statistical significance (*p* = 0.05) observed in the following studies [3, 6, 9, 10, 12, 18, 84]

^e^Results were provided overall and stratified by localized vs advanced disease at time of diagnosis. Association was observed in men diagnosed with localized disease but not advanced disease or in the study population as a whole [10, 14]

^f^Results were provided overall and stratified by localized vs advanced disease at time of diagnosis. Association was observed in men diagnosed with advanced disease but not localized disease or in the study population as a whole [10]

^g^Studies [12, 14] examined polyunsaturated *n*-3 and *n*-6 independently

^h^Statistical evidence is limited, studies only presented categorized exposure and did not provide *p* trend [22, 29, 32, 35, 59, 65, 68, 69, 83]

^i^Study [20] examined 25 OHD and 1,25(OH)_2_D; no evidence that either was associated with PCSM

^j^Study examined 25 OHD and 1,25(OH)_2_D; 25 OHD was not associated with ACM, while 1,25(OH)2D was associated

^k^Results were stratified by race (AA/B, White), statistically non-significant for both strata [26]

^l^Results were stratified by androgen dependence (independent, dependent); BMI was inversely associated with ACM among men with androgen-dependent disease but not androgen-independent disease. There was no association observed between BMI and progression in either strata [77]

^m^Statistically significant association with bone metastases but not PCSM [53, 57]

^n^Exercising is defined as METs calculated from total of walking/bicycling, household work, or exercising [41]

^o^No association observed for walking duration (hours/week) but inverse association for walking pace [42]

^p^Results were provided overall and stratified by localized vs advanced disease at time of diagnosis. Association was observed in men diagnosed with localized PC and overall, but not advanced disease [38]

^q^Heavy smoker defined as having a ≥ 20 pack-year history of smoking

^r^Results provide overall and stratified by BMI (< 25 vs ≥ 25 kg/m^2^). Association only observed among men with BMI ≥ 25 kg/m^2^ [50]

^s^Smoking cessation ≥ 10 years was not associated with recurrence/progression compared to never smokers. Smoking cessations of 1 to 4.9 years and 5 to 9.9 years were both associated with increased risk of recurrence/progression compared to never smokers [47]

## Diet

### Fish

Five studies published between 2006 and 2020 examined post-diagnostic fish intake in relation to PC outcomes, three of which considered recurrence or progression [4, 6, 7]. One of these, a study of 1202 men with non-metastatic PC from the Health Professionals Follow-up Study (HPFS), observed evidence of an inverse association in models adjusted for pre-diagnostic fish intake (hazard ratio (HR) for 1 serving/day increase: 0.52, *p* = 0.006; 95% confidence interval (CI) unavailable) [7]. The other two—a study of 940 men with stage ≤ T3 PC from Washington University and a study of 1294 men with localized/regional disease from the Cancer of the Prostate Strategic Urologic Research Endeavor (CaPSURE™)—observed no association [4, 6]. However, the Washington University study reported a statistically significant inverse association for recurrence when modeling the substitution of fish/poultry for red meat [4]. The remaining two studies examined PCSM and ACM and observed no statistically significant associations for fish intake, though one of these reported a borderline statistically significant inverse trend per 1 standard deviation (SD) of greater fish intake and ACM (HR: 0.90; CI: 0.80 to 1.01; *p* = 0.08) [3, 5]. No study reported an elevated risk of adverse PC outcomes with fish intake. In summary, evidence that fish intake following PC diagnosis is associated with PC outcomes is very limited.

### Meat, Poultry, and Eggs

Three studies conducted between 2006 and 2016 considered post-diagnostic consumption of meat, poultry, and eggs in relation to recurrence/progression and observed no associations with total poultry or total, processed, or unprocessed red meat (Table 2) [4, 6, 7]. A study of 1294 men with localized/regional PC in CaPSURE observed a positive association between poultry with skin and risk of PC progression (HR_tertile3 vs 1_: 2.26; CI: 1.36, 3.76; *p* trend = 0.003) [6]. This study also observed a borderline statistically significant association with egg intake (HR_quartile 4 vs 1_: 2.02; CI: 1.10, 3.72; *p* trend = 0.05), which was not replicated by a later study of 940 men from Washington University [4].

Three studies examined post-diagnostic intake of meat, poultry, and eggs with respect to PCSM, with mostly null results [3, 5, 8]. However, a study of 4882 men with non-metastatic PC from the Cancer Prevention Study-II Nutritional Cohort (CPS-II) reported an inverse relationship with unprocessed red meat (HR_quartile 4 vs 1_: 0.64; CI: 0.46, 0.91; *p* trend = 0.01) [3]; while a study of 926 men with non-metastatic PC from the Physicians’ Health Study (PHS) observed a higher risk per 1 SD increase in processed meats (HR: 1.32; CI: 1.06, 1.64; *p* = 0.01) [5].

Two studies examining PCSM also examined ACM, with mixed findings (Table 2) [3, 5]. In the PHS, there was a higher risk per 1 SD increase in intake of processed meats (HR: 1.17; CI: 1.06, 1.30; *p* = 0.003) and eggs (HR: 1.12; CI: 1.02, 1.24; *p* = 0.02), but no association with total red meat [5]. Conversely, the CPS-II study observed an association with total red meat (HR_quartile 4 vs 1_: 1.22; CI: 1.07, 1.39; *p* trend = 0.03), but not with eggs [3]. Furthermore, despite not demonstrating a statistically significant trend, each upper quartile (Q) of processed red meat intake in CPS-II had a higher risk of ACM compared to Q1 (HR_Q4_: 1.17; CI: 1.04, 1.33. HR_Q3_: 1.15; CI: 1.02, 1.30. HR_Q2_: 1.14; CI: 1.01, 1.28; *p* trend = 0.07) [3]. This study also reported an inverse association with total poultry and ACM (HR_Q4_: 0.84; CI: 0.75, 0.95; *p* trend = 0.01), which was not examined in the PHS.

In summary, recommendations on post-diagnostic meat, poultry, or egg intake specifically for PC outcomes cannot be made due to lack of concordance across a limited number of studies. However, based on national guidelines for general and cardiovascular health, it is prudent to limit consumption of processed meat and select lean choices of meat or skinless poultry [86, 87].

### Dairy

Three studies conducted between 2006 and 2018 considered post-diagnostic dairy intake in relation to PC recurrence or progression [7, 9, 11]. Where there was overlap in exposures examined, studies agreed. Studies in CaPSURE and HPFS both found no association with total, high-fat, or low-fat dairy, but reported positive associations between > 4 servings/week vs 0–3 servings/month of whole milk and risk of progression (HR: 1.73; CI: 1.00, 2.98; *p* trend = 0.04. HR: 1.51; CI: 1.03, 2.20; *p* trend = 0.03) [9, 11]. Two studies from the HPFS found no association between total milk and risk of PC progression [7, 11], although one of these noted a positive association in models adjusting for pre-diagnostic intake (HR_continuous_: 1.12, *p* = 0.04, CI unavailable) [7]. Table 2 shows various other dairy items that were examined by a single study [9].

Three studies examined post-diagnostic dairy intake and PCSM (Table 2) [5, 10, 11]. While results for most subcategories of dairy were null, there were consistent positive associations for whole milk. One of these, a study of 3918 men with localized/locally advanced PC from the HPFS, observed a relationship with > 4 serving/week vs 0–3 servings/month of whole milk (HR: 2.15; CI: 1.28, 3.60; *p* trend < 0.01) [11]. A population-based study of 525 Swedish men did not observe this association in the full cohort but replicated this finding for ≥ 3 vs < 1 serving/day of high-fat milk among 230 men diagnosed with localized disease (HR: 4.86; CI: 1.52, 15.57; *p* trend = 0.003) [10].

Two studies examining PCSM also examined ACM; both observed an association with high-fat dairy intake (HR_1 SD increase_: 1.18; CI: 1.07, 1.30; *p* = 0.001. HR _≥ 4.5 serv/day_ : 1.04; CI: 0.73, 1.49; HR_3–< 4.5 serv/day_: 0.82; CI: 0.58, 1.17; HR_1–< 3 serv/day_: 0.75; CI: 0.53 to 1.04 vs < 1 serv/day; *p* trend = 0.05) [5, 10]. Effect modification was observed in the Swedish cohort based on stage at diagnosis and milk type. There was a positive association for servings/day of high-fat milk (HR _≥ 3 vs < 1_: 3.32; CI: 1.85, 5.97; *p* trend = 0.001) among men diagnosed with localized PC, while low-fat milk was positively associated with ACM among 295 men diagnosed with advanced PC (HR _≥ 2 vs < 1_: 1.72; CI: 1.14, 2.57; *p* trend = 0.02) [10].

In summary, men should limit whole milk to < 4 servings/week following a PC diagnosis to minimize risk of progression and PCSM. Limiting high-fat dairy is also advised, and consistent with heart-healthy diet recommendations, to decrease risk of ACM following PC diagnosis.

### Dietary Fats

Five studies examined post-diagnostic dietary fats in relation to PC outcomes, with only one published in the last 5 years [12–16]. Only one, a study of 390 men who underwent radical prostatectomy (RP), examined risk of recurrence and reported a higher risk associated with saturated fat (HR_Q4 vs Q1–3:_ 1.90; CI: 1.16, 3.11; *p* value unavailable) [15].

Four studies examined specific types of dietary fat (Table 2) with respect to PCSM [12–14, 16]. Studies agreed that there was no association with monounsaturated, polyunsaturated, *trans*, or animal fat intake. Two studies also examined total dietary fat and found no association, although one—a Swedish study of 525 men—reported a positive trend between total dietary fat and risk of PCSM among the subgroup of men diagnosed with localized PC (HR_Q4 vs Q1_: 2.07; CI: 0.93, 4.59; *p* trend = 0.03) [14, 16]. There was mixed evidence regarding saturated and vegetable fat intake. Two studies—a Canadian study of 384 men and a study of 926 men with non-metastatic PC in the PHS—observed a relationship with saturated fat intake (HR_tertile 3 vs 1_: 3.1; CI: 1.3, 7.7; *p* trend = 0.008; HR for 5% caloric exchange of saturated fat for carbohydrates: 2.78; CI: 1.01, 7.64; *p* = 0.05) [12, 16]. Two other studies reported no statistically significant relationships between post-diagnostic saturated fat and risk of PCSM [13, 14]. Regarding vegetable fat, a study of 4577 men with non-metastatic PC from the HPFS observed an inverse relationship with PCSM (HR for 10% caloric exchange of vegetable fat for carbohydrate: 0.71; CI: 0.51, 0.98; *p* = 0.04), whereas the PHS analysis did not observe a statistically significant association [12, 13].

Two studies considered ACM with mixed results [12, 13]. Both observed no association with monounsaturated or animal fat, but an inverse association with vegetable fat (PHS HR_Q4 v Q1_: 0.65; CI: 0.45, 0.93; *p* trend = 0.03; HPFS HR_quintile5 vs 1_: 0.65; CI: 0.52, 0.83; *p* trend < 0.001) and a positive association with saturated fat (PHS HR for 5% caloric exchange of saturated fat for carbohydrate: 1.81; CI: 1.20, 2.74; *p* = 0.005; HPFS HR for 5% caloric exchange of saturated fat for carbohydrate: 1.30; CI: 1.05, 1.60; *p* = 0.02) [12, 13]. The HPFS also observed an inverse relationship between polyunsaturated fat and ACM (HR_quintile 5 vs 1_: 0.73; CI: 0.57, 0.94; *p* trend = 0.004) and a positive association with *trans* fat (HR_quintile 5 vs 1_: 1.51; CI: 1.14, 2.01; *p* trend = 0.002) [13]. The PHS observed no associations with polyunsaturated or *trans* fats [12].

Overall, diets with higher saturated fat may increase risk of PC recurrence and mortality. Replication of findings for vegetable, polyunsaturated, and *trans* fats with mortality outcomes is needed, though findings are consistent with recommendations for overall health [86, 87].

### Vegetables: Tomato (Lycopene), Cruciferous

We identified three studies that considered post-diagnostic tomato intake in relation to PC outcomes [5, 7, 17]. Two of these evaluated tomatoes in relation to risk of PC progression with inconsistent findings. The first, a study of 1560 men with non-metastatic PC from CaPSURE, found no statistically significant association with either fresh tomatoes or tomato sauce [17]. In contrast, a study of 1202 men with localized/regional PC from the HPFS reported an inverse association with tomato sauce (HR_1 serving/day_: 0.46, *p* = 0.04, CI unavailable) and a positive association with fresh tomato intake (HR_1 serving/day_: 1.27, *p* = 0.02, CI unavailable) [7]. However, associations were attenuated and neither was statistically significant when pre-diagnostic intake was excluded from the models [7]. Clinical trials have reported that supplemental lycopene is associated with return to normal PSA and normal bone scans in men with metastatic PC treated with orchiectomy [2]. Lycopene concentrations are higher in cooked than raw tomatoes, which may explain why a protective association is only observed for cooked tomatoes.

A single study of 926 men with non-metastatic disease in the PHS examined PCSM and ACM and found no association between tomato intake as part of a prudent diet and risk of PCSM or ACM [5].

Two observational studies examined cruciferous vegetables [5, 17]. CaPSURE reported an inverse association between cruciferous vegetable intake and risk of PC progression (HR_Q4 vs Q1_: 0.41; CI: 0.22, 0.76; *p* trend = 0.003) [17]. The PHS found no association with either PCSM or ACM [5].

Recent findings from the Men’s Eating and Living (MEAL) trial warrant discussion. MEAL randomized 443 men with low-risk PC on active surveillance to receive counseling promoting consumption of ≥ 7 vegetable-fruit servings/day, including at least two servings each of cruciferous vegetables and tomatoes [88]. During the 2-year intervention, 245 events of progression were observed. Though the intervention modestly increased daily servings of cruciferous vegetables (between group difference at 24 months: 0.49; CI: 0.33 to 0.64; *p* < 0.01) and tomatoes (between group differences at 24 months: 0.14; CI: 0.03, 0.26; *p* = 0.02), it did not affect risk of disease progression.

Overall, results for post-diagnostic intake of tomatoes/lycopene and cruciferous vegetables and PC outcomes are inconsistent. Nonetheless, it is prudent to encourage PC survivors to include a wide variety of vegetables in their diet for weight management and risk reduction for many chronic diseases, including diabetes and cardiovascular disease [86, 87].

### Alcohol

Two studies examined post-diagnostic alcohol consumption and PC outcomes [18, 19]. Only one, a Canadian study of 829 men with ≥ T2 disease, considered recurrence, and observed no association with total alcohol [19].

When examining PCSM, the two studies agreed there was no association for overall trend of total alcohol or liquor intake. However, the Canadian study observed a positive association with moderate intake of liquor in analyses excluding non-drinkers (HR_≥ 3.7 vs > 0–< 0.9 drinks/week_: 2.41; CI: 1.20, 4.84; *p* trend = 0.01) [19]. Conflicting findings were reported for other types of alcohol. A study of 5182 men with non-metastatic PC from the HPFS observed a borderline statistically significant positive association with beer intake (HR_≥ 7 vs 0 serving/week_: 2.64; CI: 0.58, 12.06; *p* trend = 0.05) and an inverse association with moderate total wine intake (HR_3–< 7 vs 0 serving/week_: 0.53; CI: 0.26, 1.07; *p* trend = 0.03), which appeared to be driven by red wine (HR_3–< 7 vs 0_: 0.49; CI: 0.25, 0.97; *p* trend = 0.05) [18]. Notably, the inverse association was not observed among men with higher levels of wine intake. The Canadian study found no evidence that total wine or beer were associated with PCSM [19].

Both studies also examined ACM and found no association with post-diagnostic total alcohol, beer, or liquor intake [18, 19]. However, the Canadian study observed an association with total alcohol (HR_≥ 2 vs > 0–< 2 drinks/day_: 1.45; CI: 1.06, 2.00; *p* = 0.02) and liquor (HR_≥ 3.7 vs > 0–< 0.9 drinks/week_: 1.82; CI: 1.20, 2.79; *p* trend = 0.01) in analyses excluding non-drinkers [19]. The HPFS found an inverse association with 3–< 7 vs 0 servings/week of red wine (HR: 0.64; CI: 0.45, 0.90; *p* trend = 0.007) and total wine (HR: 0.57; CI: 0.40, 081; *p* trend = 0.08), though overall trend for the latter did not reach statistical significance [18]. The Canadian study also observed an inverse association with moderate total wine intake (HR_0.2–< 0.9 vs 0 drinks/week_: 0.60; CI: 0.46, 0.79; *p* trend = 0.01) that was not observed at higher levels of wine consumption [19].

The limited data among PC survivors suggests a potential benefit of red wine at modest intake levels (1/2–1 serving/day). Men should limit total alcohol consumption to ≤ 2 drinks/day, as excess alcohol damages the heart, liver, and pancreas; increases risk of other cancers (including head and neck, esophageal, liver, and colorectal); and weakens the immune system [89]. This aligns with recommendations from many cancer control agencies [1, 90, 91].

### Supplements or Single-Nutrient Intake from Diet

#### Selenium

A single study within the HPFS examined selenium supplements (mg/day) and PC outcomes and found an increased risk of PCSM (HR_≥ 140_: 2.60; CI: 1.44, 4.70. HR_25–139_: 1.33; CI: 0.77, 2.30. HR_1–24_: 1.18; CI: 0.73, 1.91 vs 0; *p* trend = 0.001), but no association with recurrence or ACM [21].

#### Vitamin D

Three studies examined vitamin D (dietary intake or serum level) and PC outcomes [10, 20, 22]. Only one, a study of 1476 men from Seattle, examined recurrence/progression and found no association with serum 25(OH)D [22]. All three studies examined PCSM and reported no association with serum level or dietary vitamin D intake [10, 20, 22]. Two of the studies examined ACM outcomes [10, 20]. One, a study of 1119 men from New South Wales, reported an increased risk of ACM among men with higher levels of 1,25(OH)_2_D (HR_Q4 vs Q1_: 0.45; CI: 0.29, 0.69; *p* trend = 0.005) [20]. The other, a study of 525 Swedish men, observed no association between dietary intake of vitamin D and ACM [10].

#### Calcium and Phosphorous

A single study from Sweden considered both dietary calcium and phosphorous intake and observed no association with either PCSM or ACM [10].

Overall, evidence on dietary supplement use or single-nutrient intake and risk of PC recurrence or mortality is limited. Additional studies are needed to confirm the finding that selenium supplementation is associated with an increased risk of PCSM. Men with PC should follow the recommendations of the American Institute for Cancer Research and the World Cancer Research Fund and aim to meet nutritional needs through diet alone [90, 91].

## Obesity

Obesity is among the most extensively studied potential risk factor among men with PC, and the evidence is inconsistent. Regarding recurrence/progression outcomes reported between 2015 and 2020, six studies observed no association with body mass index (BMI) [25–27, 31–33], while three reported a positive association [28, 30, 34]. A report by our team attempted to clarify the discrepancies in past studies by examining adjustment for clinical and, separately, pathological characteristics in a population of men undergoing RP from CaPSURE [27]. We hypothesized that residual confounding by disease stage may partially explain positive associations reported between BMI at the time of diagnosis and risk of recurrence. We observed that with adjustment for disease severity using metrics from diagnosis (biopsy) only, there was evidence of a positive relationship between very obese men (BMI ≥ 35 kg/m^2^) and risk of recurrence [27]. However, when we controlled for surgical pathology characteristics, the observed association was no longer statistically significant. Consistent with our finding, two of the three studies that found an association between BMI and risk of recurrence did not adjust for pathologic features [30, 34]. Four of the six studies that reported no association adjusted for pathologic features [25–27, 32]. Such data suggest that obesity influences tumor aggressiveness earlier in the natural history of prostate cancer and are consistent with a larger body of evidence implicating pre-diagnosis BMI in healthy populations and risk of fatal prostate cancer [92].

Seven studies published between 2015 and 2020 examined BMI and PCSM [23, 24, 26, 29–31, 34]. Only one—a study of 1442 men treated with intensity modulated radiation therapy for localized disease, and therefore lacking pathologic measures of disease severity—observed an association (HR_continuous_: 1.15; CI: 1.07, 1.23; *p* < 0.001) [34]. Three additional studies published before 2015 also reported a positive association, only one of which controlled for pathologic metrics [57–59]. The two studies that considered waist circumference and waist-to-hip ratio found no association [23, 29].

Five studies published between 2015 and 2020 examined BMI and ACM with mixed results [23, 24, 27, 29, 34]. Three of these reported a higher risk associated with higher BMI (HR_≥ 35 vs 18.5–25_: 1.70 (1.12, 2.60), *p* trend = 0.001. HR_continuous_: 1.05; CI: 1.02, 1.08; *p* = 0.004. HR_per 5-unit_: 1.07; CI; 1.02, 1.12; *p* = 0.01) [24, 27, 34]. The two others observed no associations with BMI, waist circumference, or waist-to-hip ratio [23, 29]. There were numerous older studies that reported similarly null findings between BMI and ACM among men with PC (Table 2).

Evidence is mixed regarding if obesity measured *following* a PC diagnosis is associated with worse PC outcomes, and further research is warranted regarding whether weight loss among PC survivors who are obese offers PC-specific benefits. Nonetheless, given the relationship of obesity with other chronic diseases, including other malignancies and heart disease, men should be counseled to reach and maintain a healthy weight.

## Physical Activity

Multiple studies have examined various forms of post-diagnostic physical activity (PA) in relation to PC outcomes (Table 2). Only three of these considered recurrence/progression outcomes with mixed results [37, 40, 42]. Two examined different types of PA in the same cohort of 237 Canadian men on active surveillance [37, 40]. These studies demonstrated a lower odds of disease reclassification (OR _> 92.27 vs < 46.62_: 0.43; CI: 0.21, 0.88; *p* trend = 0.027) but not risk of progression, with higher MET-hour/week of total PA, as well as lower odds associated with vigorous PA (OR _> 0 vs 0_: 0.42; CI: 0.20, 0.85; *p* = 0.016) [37, 40]. In contrast, a CaPSURE analysis of 1455 men with localized disease found no association between vigorous PA and risk of PC progression. However, few men engaged in vigorous activity in this population, and brisk walking pace was associated with a statistically significant 57% lower risk of progression [42].

Six studies examined PA and PCSM with generally consistent findings of benefits for PA (Table 2) [35, 36, 38, 39, 41, 43]. A HPFS study of 2705 men with non-metastatic PC and a US-based study of 1354 men with localized disease reported an inverse association with vigorous PA (HR_≥ 3 vs < 1 h/week_: 0.39; CI: 0.18, 0.84; *p* trend = 0.03. HR_≥ 1 vs < 1 time/week_: 0.63; CI: 0.42, 0.95; *p* = 0.029) [36, 43]. A Canadian study of 830 men with stage ≥ T2 PC reported a 44% decreased risk for recreational PA and PCSM (> 26 vs ≤ 4 MET-hours/week, CI: 10–65%) [39]. An additional study in the CPS-II cohort similarly reported a statistically significant 31% decreased risk of PCSM associated with recreational PA [38]. A 2015 study of 4623 Swedish men with localized PC reported a 32% reduction in risk of PCSM for ≥ 1 vs < 1 h/week of exercise after diagnosis (CI 6–52%); a similar benefit was reported for walking/biking ≥ 20 vs < 20 min/day, but not for total recreational physical activity or household work [41]. While there has been variability in the type, duration, or intensity of PA associated with PCSM benefits, these reports suggest that PA offers benefit for reducing risk of PCSM.

Five studies examining PCSM also examined ACM and overwhelmingly reported an inverse relationship with PA [35, 38, 39, 41, 43]. The risk reduction comparing the highest to lowest PA categories were as follows: 42–62% for total PA, 35–49% for vigorous PA, 14–37% recreational PA, and 7–30% for walking/biking [38, 39, 41, 43].

In summary, there is strong evidence that increased PA following PC diagnosis is associated with lower risk of PCSM and ACM. The 2018 National PA Guidelines in the USA recommend that adults do ≥ 150 min/week of moderate-intensity or ≥ 75 min/week of vigorous-intensity aerobic PA. These guidelines report lower risk of PC mortality as a health benefit associated with regular PA for PC survivors [93•]. In addition, clinical trials have shown that PA improves bone mineral density and quality-of-life among men undergoing androgen deprivation therapy for PC [2•]. Considering the totality of evidence, we recommend that PC survivors engage in regular PA. Trials are underway to develop interventions to help men with PC meet PA goals, while considering a man’s current capabilities and health-related concerns (see Table 2 in ref. [2•]).

### Smoking

Multiple studies have examined the relationship between smoking and PC recurrence/progression and PCSM (Table 2). There is overall agreement that men reporting smoking following diagnosis are at higher risk of recurrence/progression and PCSM compared to never smokers [44, 46, 47, 51–53, 55].

Some evidence exists that the duration of smoking cessation may affect the risk of PC outcomes among former smokers. Specifically, an Austrian study of 6538 men with localized PC reported that former smokers who had quit ≥ 10 years prior had a similar risk of recurrence as never smokers, but those who had quit < 10 years prior were at increased risk of recurrence [47]. Results from a US-based study of 752 men for the outcome of PCSM support this conclusion, though results did not reach statistical significance [52]. Limited data on former smoking duration and dose may account for the mixed evidence regarding whether former smokers are at an increased risk for poor PC outcomes [44, 46, 47, 51–54].

Fewer studies examined ACM outcomes [44, 48, 53, 56]. A Canadian study of 434 men with localized disease found no association between former or current smokers and ACM, though it was limited by a short follow-up period (median 70 months) [53]. As expected, all other studies found a statistically significant increased risk of death associated with smoking [44, 48, 56].

In summary, current smokers are at an increased risk of disease recurrence/progression, PCSM, and ACM. Men who smoke should be provided with resources to help them quit to improve their PC-specific prognosis and overall health.

## Diversity of Study Populations

### Race/Ethnicity

AA/B men experience higher rates of PC incidence and mortality than men of any other race/ethnicity. In the USA, the rate of PCSM is more than twofold higher in AA/B vs White men (40.8 vs 18.2 per 100,000 in AA/B and White men, respectively) [94]. Despite this fact, existing evidence on post-diagnostic modifiable risk factors has been collected almost exclusively in White populations. Characteristics of 33 recently published (2015–2020) studies are shown in Table 1; 13 did not report the racial/ethnic distribution of their study sample [10, 19, 21, 29, 32, 34, 35, 37, 38, 41, 45–47]. An additional seven dichotomized race as White/Caucasian vs other (all ≥ 92% White) [4, 5, 12, 18, 24, 31, 39]. Only six included ≥ 10% AA/B/African-Caribbean men [23, 26, 28, 30, 33, 44].

Few studies have examined whether the associations between lifestyle factors and risk of PC outcomes vary by race/ethnicity. The two that provided results stratified by race (AA/B vs White) both examined BMI as the primary exposure [26, 33]. The first was a study of 5929 (33% AA/B) men treated via RP that observed no association between BMI and PCSM or recurrence, overall or in either race/ethnicity stratum [26]. The other was a study of 647 men that reported a positive association between BMI ≥ 30 vs BMI < 30 with PC recurrence among the 363 White men (HR: 1.80; CI: 1.09, 2.96) but not among the 284 AA/B men (HR: 1.10; CI: 0.69, 1.76) [33].

Although limited, a few studies have identified mortality disparities among other underrepresented racial/ethnic minority populations. For example, Puerto Rican and Mexican American men may have an increased risk of PCSM compared to White men [95, 96]. Future studies should report race/ethnicity for their study population and test for effect modification by race/ethnicity when numbers allow. Deliberate and targeted recruitment of AA/B men and other high-risk populations into PC-related studies is crucial. In the interim, it should be a priority to identify existing data sources with a sufficient proportion of AA/B men and other underrepresented racial/ethnic minorities to begin to address these questions.

### Education

We intended to examine educational attainment as a measure of socioeconomic status; however, only 10 of the 33 recent studies (2015–2020) reported education levels of their study populations [3, 9, 19, 23, 24, 33, 35, 36, 39, 41].

### Cohorts

Most of what we know regarding diet and lifestyle following a PC diagnosis comes from a limited number of cohorts. Table 1 displays literature by exposure, consisting of 64 (non-unique) studies. The HPFS, CaPSURE, and PHS-II account for one-third (*n* = 22) of these (note, EVB, SAK, and JMC were authors on many of these papers). An additional 15% (*n* = 10) are from CPS-II, the Shared Equal Access Regional Cancer Hospital (SEARCH) database, and Royal Marsden Hospital. Finally, many of the exposures were examined by only a single study (Table 2), highlighting areas where replication and confirmation is needed.

### Future Direction

In summary, research to date on post-diagnostic lifestyle factors and risk of PC recurrence and mortality has been limited to a few cohorts of predominately White men. Large cohorts that are racially/ethnically, geographically, and socio-demographically diverse are necessary to advance this field of research.

## Conclusions

In this review, we focused on observational evidence of post-diagnostic modifiable diet and lifestyle factors in relation to PC outcomes. Though randomized trials are the gold standard for determining causation, many diet and lifestyle behaviors are not suitable/ethical (e.g., smoking) to randomization. Furthermore, long-term and slow-acting exposures may require extended follow-up periods to observe outcomes of interest, which may preclude study in a randomized setting. Overall, the evidence reviewed suggests that following a PC diagnosis, men should be counseled to increase physical activity and quit smoking, consistent with general health recommendations. Additionally, it may be prudent for men with PC to minimize whole milk/high-fat dairy intake; for those who consume alcohol, consider moderate consumption of red wine (e.g., ½ to 1 glass/day) over other types of alcohol, and aim to meet nutritional needs through food rather than supplements. Future research that includes more diverse populations, particularly AA/B men, is needed.
